# Design of Under-Actuated Soft Adhesion Actuators for Climbing Robots

**DOI:** 10.3390/s22155639

**Published:** 2022-07-28

**Authors:** Zhipeng Liu, Linsen Xu, Xingcan Liang, Jinfu Liu

**Affiliations:** 1 Hefei Institutes of Physical Science, Chinese Academy of Sciences, Hefei 230031, China; liuzhipeng@mail.ustc.edu.cn; 2 University of Science and Technology of China, Hefei 230026, China; lxcan@mail.ustc.edu.cn; 3 College of Mechanical and Electrical Engineering, Hohai University, Nanjing 210098, China; 4 Suzhou Research Institute, Hohai University, Nanjing 210098, China; 5 Changzhou Vocationnal Institute of Industry Technology, Changzhou 213018, China; liujinfu@mail.ustc.edu.cn

**Keywords:** under-actuated soft adhesion actuator, load-capacity climbing device, controllable adhesion, adaptability to various surfaces

## Abstract

Since climbing robots mainly rely on adhesion actuators to achieve adhesion, robust adhesion actuators have always been the challenge of climbing robot design. A novel under-actuated soft adhesion actuator (USAA) proposed in this paper for climbing robots can generate adhesion through robot’s load applied to the actuator. The actuator is composed of a soft film/substrate structure with an annular groove on the substrate and a cavity on the soft film. To fabricate the actuator, we first study the influence of the geometric parameters of the USAA on the maximum adhesion of the actuator by analysis and experiments, and then combine these parameters and the boundary conditions of the static meniscus in the mold to design the mold. Moreover, we fabricate a climbing robot equipped with USAAs and evaluate its performance on horizontal and inclined surfaces with a wide range of characteristics. The USAA can generate strong and controllable adhesion to various smooth and semi-smooth surfaces. Furthermore, the fabricated robot performs well on various surfaces under a certain load (at least 500 g) and speed (369 mm/min) through experiments. It’s adaptability to a variety of surfaces enables a wide range of applications and pushes the boundaries of soft adhesion actuators.

## 1. Introduction

As an important direction for the development of robots in three-dimensional space operations, climbing robots have a wide range of potential applications, including the cleaning, monitoring, and maintenance of high-rise buildings and bridges, etc. [1,2]. The stable work of the climbing robot mainly depends on the reliable attachment ability. Bioinspired by the adhesion systems of insects and animals in nature like gecko feet [3,4], files [5], inchworm [6], octopus [7,8], clingfish [9], and numerous studies have focused on mimicking their adhesion mechanism. In addition, the effective locomotion modes not only ensure that the climbing robot adapts to different working environments, but also has low energy consumption. Furthermore, in order to further improve the adhesion control, studies focused on safety and reliability of wall-climbing robots, wall-cleaning robots with adhesion awareness [10,11], and wall-climbing with safe navigation [12]. The locomotion modes are mainly divided into four categories such as foot type [13,14], crawler type [15,16], wheel type [17], and translation type [18].

At present, a variety of classic corresponding adhesion modes have been developed for specific environments, mainly including vacuum, magnet, adhesion, and Vortex [19], etc. Among them, there are two main mechanisms for non-metal surface adhesion pads: dry adhesion inspired by gecko foot-bristles [20] and vacuum suction adhesion inspired by octopus [21,22] and clingfish [23]. However, due to the lack of self-cleaning ability, the current dry adhesion cannot adapt to dirty and smooth surfaces. Among these robots, wall-climbing robots with wet adhesion systems have some potential to work on rough surfaces [24]. In addition, because the vacuum suction cup is made of very soft materials, it is easy to cause structural failure [25,26] and can not meet the strong and stable adhesion required by the climbing robot during operation. At the same time, the accessory vacuum pump and gas pipeline of the vacuum suction cup add extra load and weaken the working ability of the climbing robot. In Ref. [27], the passive suction cup can well realize the basic functions of the wall-climbing robot, but its adhesion is not controllable.

Although the current attachment technology is very advanced, more convenient and faster attachment structures and methods need to be further explored, which will help to broaden the functional boundaries of climbing robots. Negative pressure attachment is the most common attachment mode in nature, and its wide potential application value has always been the focus of researchers in the field of bionics and robotics, and it has also achieved good results in practical applications. Furthermore, the versatility of the climbing robot largely depends on its load capacity. In order to increase the load capacity of the climbing robot, the main challenge lies in the design of the fast-switchable adhesion actuator with strong adhesion. This not only enables the climbing robot to perform climbing operations quickly and efficiently, but also makes it easier for climbing robots to operate in complex environments.

Here, we propose a novel under-actuated soft adhesion actuator (USAA) for climbing robot, which can carry a considerable payload in multi-mode locomotion to address the above challenges. The actuator is made of highly elastic soft material without corresponding pneumatic channel, and the risk of structural failure is considerably smaller. Different from the traditional vacuum suction formed by negative and positive pressure deformation, we use the external load to deform the double-layer structure of the actuator to form a vacuum chamber to achieve robust and efficient adhesion force. The following sections provide details about the concept, manufacturing, experimental setups, and results of the actuator and climbing robot characteristics. Section 2 describes the working mechanism of under-actuated adhesion actuator, and provides the details of adhesion actuator design, including the essential geometric parameters and fabricating mechanisms. Section 3 shows the performance of a climbing robot equipped with adhesion actuators through experiments. Discussion and conclusions are presented in Section 4.

## 2. Under-Actuated Adhesion Actuator Design

**Figure 1:**
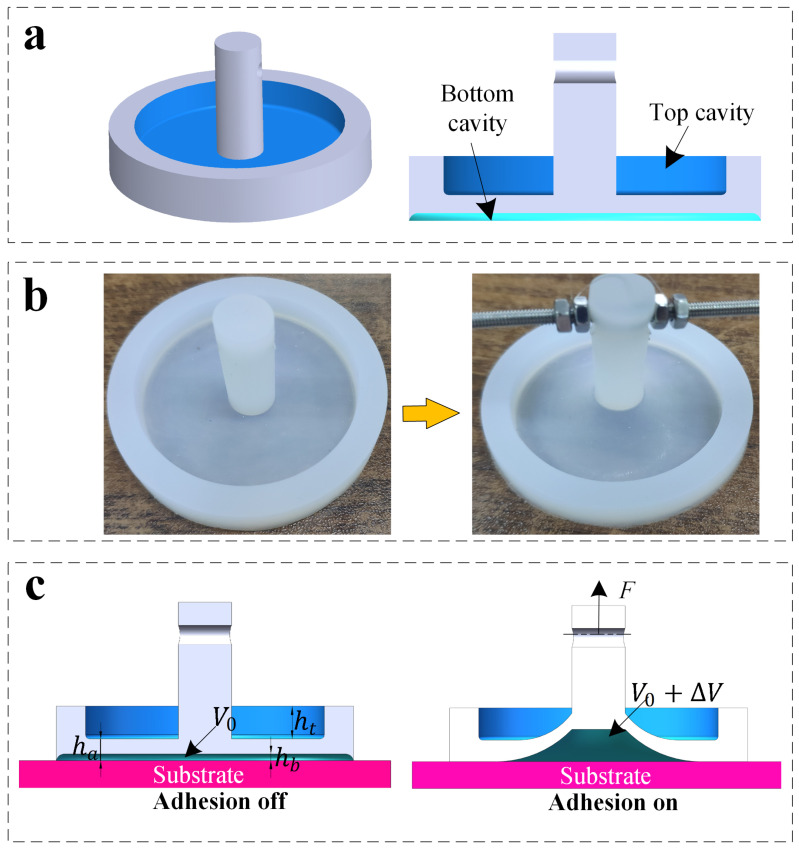
Structure and design of soft adhesion actuators. (**a**) structural design of double-layer actuator with ring groove on the top and cavity underneath; (**b**) The prototype actuator (left) deforms into a dome shape after applying a vertical load (right); (**c**) schematic of the operation for actuator upon external load; adhesion-off state (left), adhesion-on state (right).

**Figure 2:**
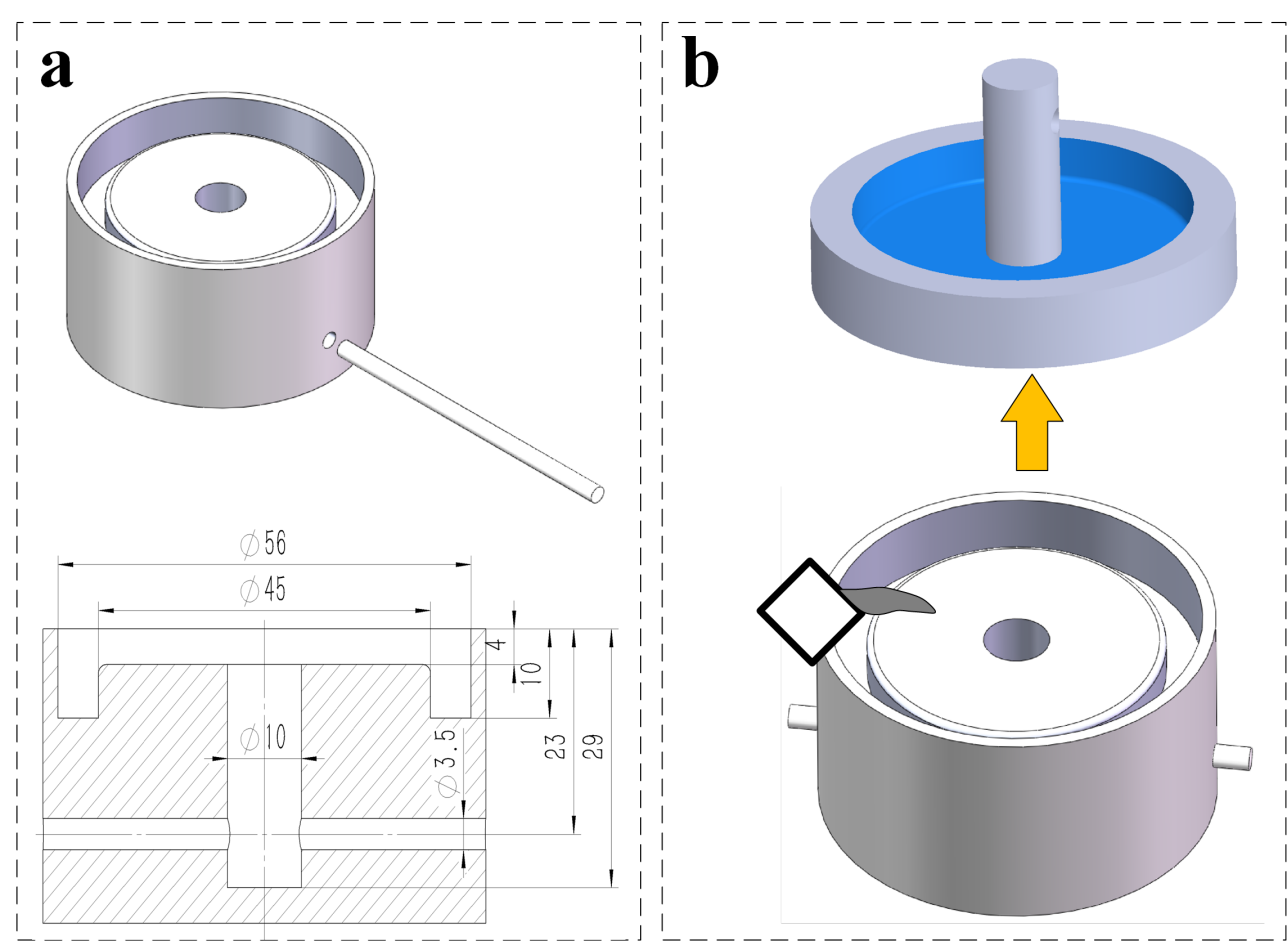
Schematic of fabrication of the soft adhesion actuator.

Figure 1a shows the design schematic of under-actuated adhesion actuator. It is a film/substrate structure consisting of a ring groove on the top and a meniscus cavity formed naturally after casting on the bottom. In addition, the height of the meniscus cavity is extremely small compared to the height of the entire actuator. To fabricate the USAA, we use a 3D printed mold, which includes annular groove and cylindrical hole as shown in Figure 2a. The elastomer actuator (white, similar Ecoflex 00-50; Smooth-on, Inc, Macungie, PA, USA) is cured in this mold for four hours at room temperature. The fabricated actuator after demolding is shown in top of Figure 2b. The state of the actuator before (left) and after (right) loading the payload on the prototype actuator as shown in Figure 1b, respectively.

The adhesion mechanism of the soft actuator is schematically illustrated in Figure 1c. Rather than squeezing air out of the cavity like traditional sucker, we apply the load on the top of the actuator to generate negative pressure in cavity for adhesion. When the vertical load is applied to the top, the mismatched expanding deformation will occur in the film/substrate structure of actuator, and its cavity forms a dome shape as shown on the right of Figure 1b,c [28]. After deforming into a dome shape, the volume of the cavity increases to $V_{0}+\Delta V$( $V_{0}$ and ΔV are initial volume and the volume change of the cavity, respectively). Before the load is applied, the air pressure in the cavity is balanced with the external atmospheric pressure. When the load is applied, according to the ideal gas law $P_{0}V_{0}=\left(P_{0}-\Delta P\right)\left(V_{0}+\Delta V\right)$, the volume of the cavity increases and the pressure drop inside the cavity causes a pressure difference between the inside and outside of the cavity, which makes the actuator stick to the target surfaces. The adhesion force is determined by the pressure difference, which can be adjusted by the cavity volume and other geometric parameters including the depth of groove $h_{t}$ and the layer thickness above the cavity $\left(h_{a}-h_{b}\right)$, as shown in Figure 1c.

It is simple for us to switch the actuator rapidly and reversibly by loading and unloading load, respectively. The traditional pneumatic actuators use pumps directly to pump out or inject air to generate positive pressure or negative pressure to obtain adhesion force. However, the adhesion force generated by negative pressure is limited by the maximum pressure difference [26], and there is a potential risk of structural rupture because of extreme deformation [29,30]. Compared with the negative pressure-based actuators, the positive pressure actuators are more stable, but the actuator structure will deform unpredictably when pumping air into the cavity. Furthermore, the pneumatic drive also reduces the execution rate of the actuator [29]. In contrast, the adhesion force of under-actuated adhesion actuator is much more stable under the external load. When the preload is applied to the actuator, the structural stability of the soft adhesion actuator can also be strengthened.

### 2.1. Modeling Adhesion Actuator as Double-Layer Doming System

In order to explain the design of the actuator, the approximate film/substrate model with a cylindrical groove is employed. In addition, the mismatch strain between the film and the substrate of the simplified thin film/substrate system can explain the change in the cavity of adhesion actuator after the load is applied.

**Figure 3:**
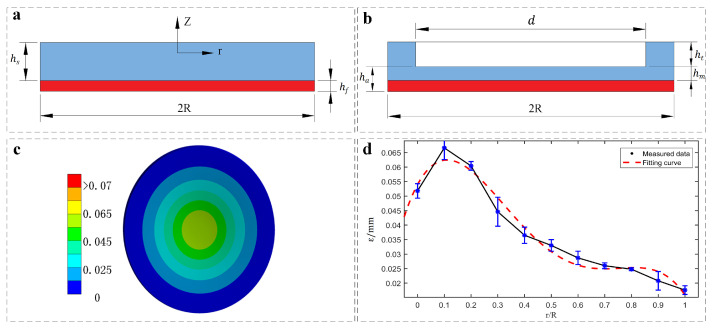
(**a**) Schematic of thin film (red)/substrate (blue) system with misfit strain for doming deformation; (**b**) schematic of the proposed bilayer doming model with cylindrical groove; (**c**) The finite element analysis result of the actuator upon 1 kg payload shows the radial strain; (**d**) the measured strain of the actuator along the radial direction.

The film/substrate system with length *R* in the lateral direction is composed of a thin film (thickness of $h_{f}$) deposited on a more thicker substrate (thickness of $h_{s}$), as shown in Figure 3a ($R\gg h_{s})$. When the radially symmetric force field is applied to the film/substrate system, a mismatch strain ε(r) along the radial direction *r* will be generated. Then, the displacement *w* of this deformed structure along the *z*-axis direction can be expressed simply as [31]:

(1) $$\begin{array}{cc} w & =-\frac{6E_{f}h_{f}}{1-v_{f}^{2}}\frac{1-v_{s}^{2}}{E_{s}h_{s}^{2}}\left(1+v_{s}\right)[\int_{0}^{r}\frac{1}{r}\int_{0}^{r}\eta \varepsilon \left(\eta \right)d\eta \\ \; & +\frac{1-v_{s}}{1+v_{s}}\frac{r^{2}}{2R^{2}}\int_{0}^{R}\eta \varepsilon \left(\eta \right)d\eta ]+C \end{array}$$

where the subscripts “*f*” and “*s*” represent film and substrate, respectively. *E*, ν, and *r* denote the Young’s modulus, Poisson’s ratio, and radial coordinate. When we assume boundary conditions that the force and moment are vanish (near the edge r=R), that is w(R)=0, *C* is a constant parameter, and w(R)=0 needs to be satisfied. As the film/substrate system is applied to the actuator, we can obtain the schematic diagram as shown in Figure 3b. Furthermore, we use $\left(h_{a}-h_{m}\right)$ and $\left(h_{t}+h_{m}\right)$ instead of $h_{f}$ and $h_{s}$. Because the two layers are made of the same material, which is hyperelastic body, the material properties of *E* and ν are unified. Therefore, the double-layer model employed can be simplified and the groove in the substrate can be ignored, when taking account of the effect of the mismatch expansion caused by the load. Here, incorporating the above conditions into Equation (1), the *w* is given by:

(2) $$\begin{array}{cc} w & =\frac{dw}{dr}=-6h_{f}\frac{1+v}{h_{s}^{2}}[\int_{0}^{r}\frac{1}{r}\int_{0}^{r}\eta \varepsilon_{m}\left(\eta \right)d\eta dr\\ \; & +\frac{1-v}{1+v}\frac{r^{2}}{2R^{2}}\int_{0}^{R}\eta \varepsilon_{m}\left(\eta \right)d\eta ]+A \end{array}$$

Based on expansion of the system, the volume change of the bottom cavity can be expressed as follows:

(3) $$\Delta =\frac{1}{3}\pi \int_{0}^{R}w\left(R^{2}+A+RA\right)dr$$

Compared with the displacement along the radial direction, the diameter of the load application position and the wall thickness of the cavity are large enough to ignore the radical displacement in Equation (3), where *A* is the constant diameter of the bottom surface of the actuator. Furthermore, Equation (3) is not suitable for pressure change caused by gas infiltration and volume change. In the future, taking the relationship between tension and volume change into account, a more detailed and accurate model can be established.

Combining the ideal gas law with Equation (3), the pressure difference applied to the actuator can be obtained as follows:

(4) $$\Delta P=\frac{P_{0}\Delta V}{V_{0}+\Delta V}$$

According to the above equations, the pressure change of the cavity can be predicted with the film/substrate system. The strain ε caused by radially symmetric force fields F(r) applied to the film/substrate system plays an important role in pressure change.

We use finite element analysis to collect the expansion data of the actuator to quantify the functional relationship between the strain ε of the actuator along the radial direction and r upon load. Figure 3c shows a finite element strain image on the adhesion actuator, where the measured strain parameters as a function of the radial position upon 1 kg payload are plotted in Figure 3d. The result of analysis indicates that ε exhibits obvious nonlinear variation along the radial direction upon constant payload, where the strain arrives its peak at r/R≈0.1 and reaches a minimum at the edge of the soft actuator. In addition, the result also conforms to the high-order nonlinear relationship between stress and strain under the condition of large deformation of the super elastic body. Then, when the fitting curve of the finite element analysis data represented by $\varepsilon =-0.67t^{4}+1.457t^{3}-0.9t^{2}+0.169t+0.05$ (the red dashed line, t=r/R) is submitted into Equation (2) and (3), we can predict the theoretical volume change of the actuator upon load as $\Delta V/V_{0}=0.16$, which has a good agreement with the analysis result upon 1 kg load: the error range is ±0.02.

### 2.2. Essential Geometric Parameters in the Adhesion Actuator

The proposed adhesion actuator is compliant, and its adhesion performance mainly depends on shear adhesion, which is along the tangent direction of the contact surface and is influenced by a couple of geometric parameters. Here, for an adhesion actuator with a given outer diameter *R* and height *h*, the diameter *d* of the groove and the layer thickness $h_{m}$ between the groove and the bottom cavity (Figure 3b) have a decisive influence on the volume change of the cavity deformation. Since the volume of the silica gel does not change after deformation, the simplified Laplace relationship between the pressure in the deformed cavity and the expansion coefficient in this thin wall system is as follows [32]:

(5) $$P=4\omega \frac{h_{0}}{r_{0}}\left(\lambda^{-1}-\lambda^{-7}\right)\;(N/\mathrm{mm}{}^{2})$$

where $r_{0}$ is the initial radius of the cavity and λ is the expansion coefficient, which is different from the strain ε. The pressure *p* in the deformed cavity reaches a maximum at λ=1.38; (here the expansion coefficient λ<1 is maintained to balance the system deformation and adhesion force).

In addition, the vertical and lateral stiffness also have an effect on the expansion deformation [33]:

Vertical stiffness:

(6) $$k_{v}=\frac{A_{c}\mu_{1}E}{h}\;\left(N/\mathrm{mm}\right)$$

Lateral stiffness:

(7) $$k_{L}=\frac{A_{c}G}{h\left[1+\frac{4}{9}{\left(\frac{h}{2R}\right)}^{2}\right]}\;\left(N/\mathrm{mm}\right)$$

The ratio of Equation (7) to Equation (6) is:

(8) $$\tau =\frac{k_{v}}{k_{L}}=\frac{\mu_{1}E\left[1+\frac{4}{9}{\left(\frac{h}{2R}\right)}^{2}\right]}{G}<1,$$

where $\mu_{1}$ is the vertical shape factor, *E* is Young’s modulus, and *G* is the shear modulus.

It can be seen from Equation (8) that the lateral stiffness of the system is greater than the vertical stiffness, so the vertical deformation of the system is more obvious than the lateral deformation when the bottom cavity expands and deforms.

**Figure 4:**
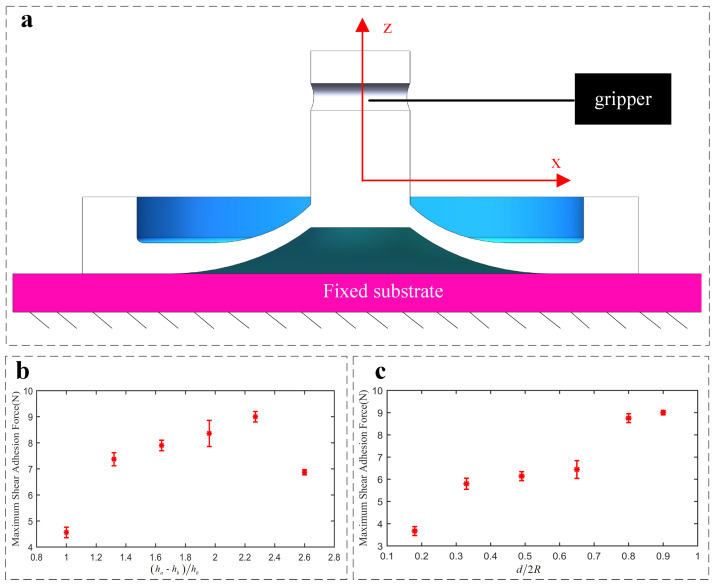
(**a**) Schematic of the shear adhesion force test of the actuator; (**b**) the measured maximum shear adhesion force (payload weight 500 g) attached to acrylic surfaces by varying d/2R$((h_{a}$ − $h_{b}$)$/h_{b}=2.27,h_{b}=1.22)$ as illustrated in Figure 1c; (**c**) the measured maximum shear adhesion force (payload weight 500 g) attached to acrylic surfaces by varying $(h_{a}$ − $h_{b})/h_{b}$(d/2R=0.8) as illustrated in Figure 1c.

**Figure 5:**
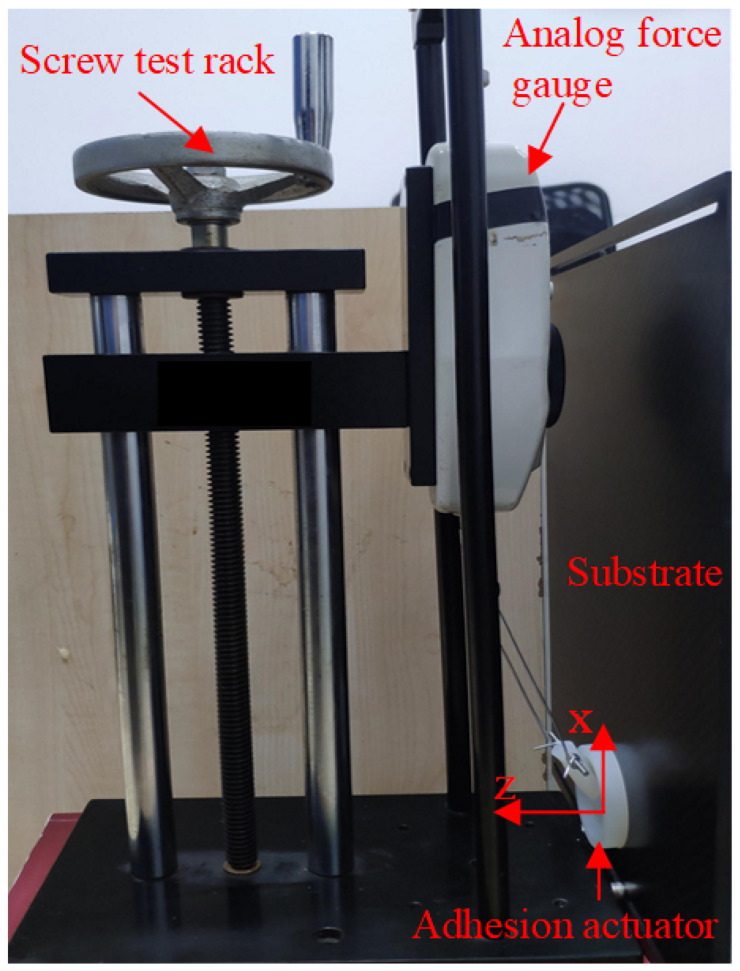
Experimental testing of maximum shear adhesion force.

Furthermore, the performance of the adhesion actuator can be quantified by the maximum tangential adhesion force $F_{S}$, which is defined as the critical shear load when the actuator is detached or slides on the surface of attached acrylic board and can be measured as shown in Figure 4a. In order to quantify the performance of the adhesion actuator, we conduct shear adhesion force test by using an analog force gauge (Aidebao Co., Wenzhou, China) with a range of 500 N along the *x*-axis at the speed of 1 mm/s on the screw test frame, as shown in Figure 5.

In order to study the influence of these parameters on the tangential adhesion of the soft adhesion actuator, the same property of carbon fiber board and payload 1 kg are employed. The results of the parameter analysis (Figure 4b,c) show that appropriate values of $h_{a}/h_{m}$ and d⁄2R are conducive to obtaining strong and robust adhesion.

Figure 4b illustrates that $F_{S}$ and the value of $(h_{a}$ − $h_{b})/h_{b}$ are nonlinearly correlated. As the value of $(h_{a}$ − $h_{b})/h_{b}$ increases, $F_{S}$ increases to its maximum at $(h_{a}$ − $h_{b})/h_{b}=2.27$ and then shows a downward trend. When $(h_{a}$ − $h_{b})/h_{b}$ is close to 1, the stiffness of soft actuator is smaller and the radial expansion of the actuator structure dominates the deformation rather than the cavity bulge. On the contrary, if the value of $(h_{a}$ − $h_{b})/h_{b}$ becomes too large, the increased vertical stiffness of cavity will lead to weak adhesion upon the same payload. Thus, $(h_{a}$ − $h_{b})/h_{b}=2.27$ is chosen for the largest tangential adhesion.

As mentioned above, the greater the volume change of the bottom cavity, the stronger adhesion force that is achieved by the soft actuator. Therefore, when the height of the bottom cavity is zero, the theoretical adhesion force can reach its peak. However, upon actuation, it is difficult for a soft actuator without bottom-cavity to firmly adhere to the surface of substrate because there is potential for air leakage without an airtight skirt. Therefore, the natural cavity formed by casting can maintain the balance between the consistency and adhesion of the actuator. In addition, there is a slight difference in the rising height of liquid column in the vertical circular mold due to the difference in the volume of cast mold and the contact-angle hysteresis (here the height of bottom-cavity is 1.22±0.02 mm).

Furthermore, in order to improve the adhesion force, we can adjust the ratio of the top-groove diameter to the outer diameter of the actuator d/2R. Since the outer diameter of the actuator is fixed, here we mainly adjust the diameter of the top groove in the same unit volume. While keeping other parameters constant, a larger groove results in larger $F_{S}$. We can explain it as follows: a relatively larger groove diameter *d* can lead to larger value $\Delta V/V_{0}$ because of greater flexibility along the radial direction of the actuator. Upon Equations (2) and (4), as *d* increases, the higher $F_{s}$ can be obtained. However, when the *d* becomes too large, it is difficult for the actuator to form a stable airtight skirt. In addition, because of potential air leakage, it will also cause weak adhesion, especially on semi-smooth target surfaces. Thus, here we chose d/2R=0.8 to balance the conformability and adhesion force of the actuator itself.

The design of the adhesion actuator is inspired by the shrinkage phenomenology of liquid silicone during the curing. We employ the CSF model to explain the cavity of the adhesion actuator formed by the adhesion force of the mold wall [34].

**Figure 6:**
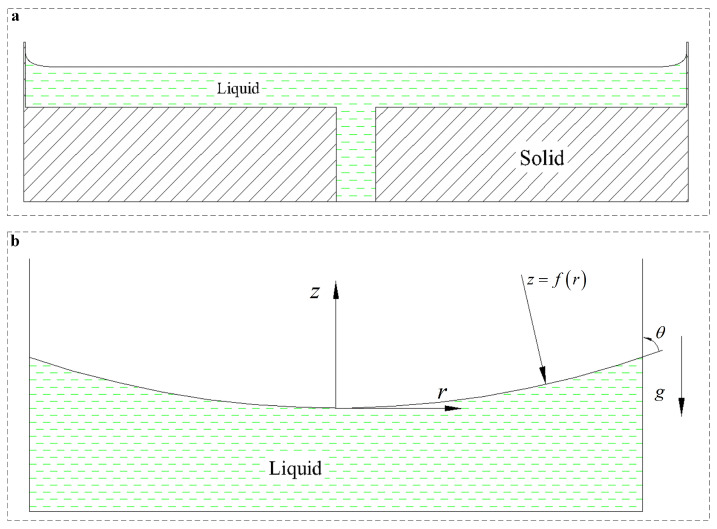
(**a**) The casting model; (**b**) physical parameter configuration of the model.

It is used to cure liquid silicone in a vertical right circular cylinder mold with inner radius of *R* as illustrated in Figure 6a. When it is affected by the surface tension and the adhesion force of the mold wall, the flow field causes the liquid silicone to move upward along the wall (Figure 6b) until the following equation is satisfied [35,36,37]:

(9) $$\begin{array}{c} \frac{1}{r}\frac{d}{dr}\left[\frac{rdf(r)/dr}{{\left[{\left(1+df\left(r\right)/dr\right)}^{2}\right]}^{\frac{1}{2}}}\right]-Bf\left(r\right)=0\;\left(0<r<1\right) \end{array}$$

with the boundary conditions

(10) $$\left\{\begin{array}{cc} df(r)/dr=0, & r=0\\ df(r)/dr=cot\theta , & r=1\\ f(0)=0 \end{array}\right.$$

where $B=\rho \alpha R^{2}/\sigma$ is the Bond number with the density ρ, the acceleration of gravity *g*, the inner radius of the cylinder mold *R*, and the critical surface tension of liquid silicone σ, Rr and Rf(r) represent the radial distance from the axis and the height of surface, respectively; and $\theta_{}$ is the contact angle between the liquid silicone and mold wall.

Furthermore, the boundary conditions of wall adhesion should also be considered, which can be expressed as [38]:

(11) $$\hat{n}={\hat{n}}_{wall}cos\theta_{eq}+{\hat{n}}_{t}sin\theta_{eq}$$

where ${\hat{n}}_{wall}$ is located on the mold wall and perpendicular to the line of intersection between the interface and the mold wall, and ${\hat{n}}_{t}$ is the normal line of the wall element pointing to the mold wall. The mold is wetted by liquid silicone; in that case, $\theta_{eq}$ is the static equilibrium contact angle with the value of $5^{\circ }$ [39].

These boundary conditions applied along the normal of the mold wall lead to upward surface force, which creates a pressure field close to the wall. Substituting $\theta_{eq}$ and $h_{b}$ into Equations (9) and (10), we can calculate the inner radius of the cylinder mold *R* as shown in Figure 1a.

## 3. Climbing Robot with Soft Adhesion Actuators

As mentioned above, the soft adhesion actuator with strong and robust adhesion force has been designed. Then, we will combine the adhesion actuators and the leg-type device to construct a climbing robot that can locomote on the ground to demonstrate work performance.

**Figure 7:**
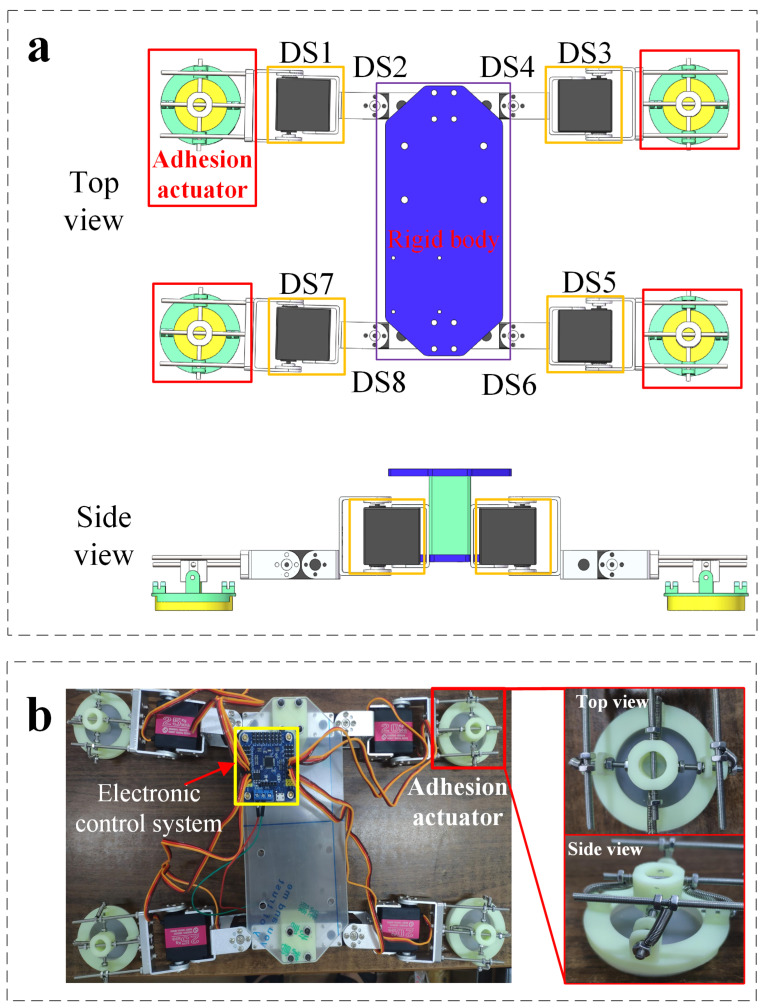
Design of climbing robot. (**a**) top view (bottom) and side view (top) of the schematic design of the climbing-robot composed of adhesion actuators (on the ends of the four legs with four digital servos:DS1, DS3, DS5, DS7) for switchable adhesion, and one rigid body (equipped with four digital servos: DS2, DS4, DS6, DS8) for locomotion driven by digital servos; (**b**) the initial state of fabricated climbing-robot from the design in (**a**).

**Figure 8:**
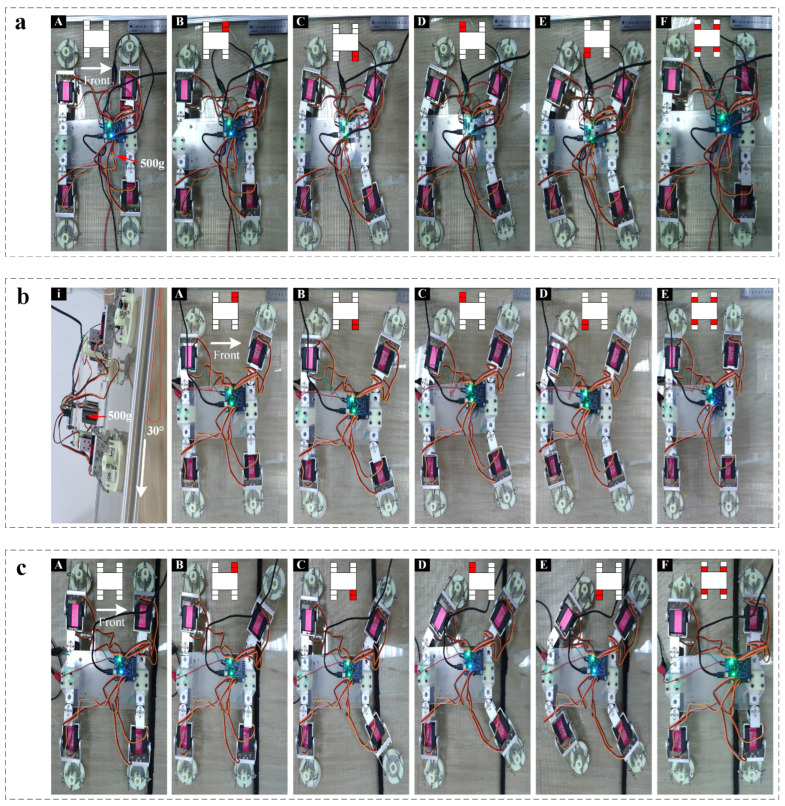
Demonstration of the walking and climbing mode of climbing-robot with a carried load on ground. (**a**) walking mode on a smooth acrylic surface with a carried load of a 500 g weight by actuating digital servos (**A**–**F**). In the top middle inset, the red color indicates digital servos activated while the white color represents the inactivation, correspondingly; (**b**) demonstration of the sequential actuating digital servos (**A**–**E**) for climbing on an 30° inclined acrylics surface with a carried load of 500 g; (**c**) demonstration of the sequential actuating digital servos (**A**–**F**) for overcoming obstacle (the height is 16 mm the width is 10 mm).

In previous studies, there are two main implementation methods for the leg-type climbing robots: quadruped robot with adhesion actuators on the feet [13], or crawler robot with vacuum pad array evenly distributed along the length of track [15,16]. In this research, our design of climbing robot is inspired by the locomotion of gecko, as illustrated in Figure 7a. Similar to the gecko, our fabricated climbing robot consists of four legs with end-adhesion actuators and a rigid body, as shown in Figure 7b. The initial length and width of the robot are 15 cm and 18 cm, respectively, and each leg is 5 cm long. Each leg has two active degrees of freedom driven by the digital servo and two passive degrees of freedom on the end adhesion actuators (Figure 8a), which enable adaptive adhesion demanded for attachment and separation from the target surfaces.

**Figure 9:**
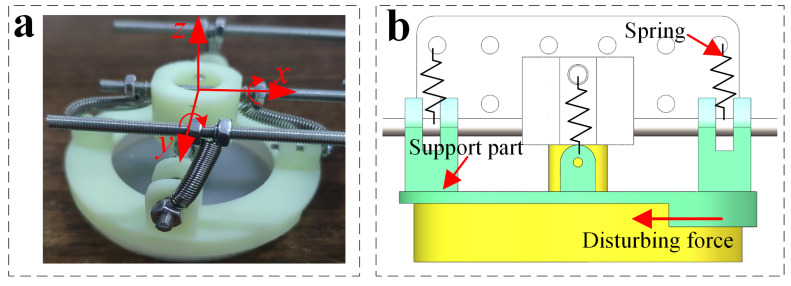
The structure of robot’s foot. (**a**) The adhesion actuator can rotate around the *x*-axis and *y*-axis; (**b**) illustration of the disturbing force of the support part (green) to adhesion actuator (yellow).

By actuating the four legs in sequence with the electronic control system (Figure 7b), we exhibit the walking (Figure 8a) and climbing (Figure 8b) modes of the designed robot on a smooth surface (e.g., acrylic plate) with a certain load capacity. The process of interaction between the adhesion actuator and the target surface in a crawling cycle can be divided into three sequential phases: pre-tensioning phase, adhesion phase, and de-adhesion phase. At the pre-tensioning phase, force is applied to the adhesion actuator along the *z*-axis direction (Figure 9a) to make the bottom surface fully fit the target surfaces. In addition, as shown in Figure 9a, the two DOFs of the robot foot end are the rotational DOF of the *x*-axis and the *y*-axis, respectively. Increasing the direct contact area between the bottom surface of the adhesion actuator and the target surface can prevent air leakage, and increase the friction between the actuator and the target surface. At the adhesion phase, when the leg is lifted in the direction perpendicular to the contact surface, the actuator deforms to generate the corresponding adhesion force. At the third phase, lower the leg to restore the deformation of actuator. Then, when the leg swings forward, the support part of the actuator interferes with the actuator in the radial direction to destroy its air tightness, which causes the actuator to detach from the target surfaces (Figure 9b).

**Figure 10:**
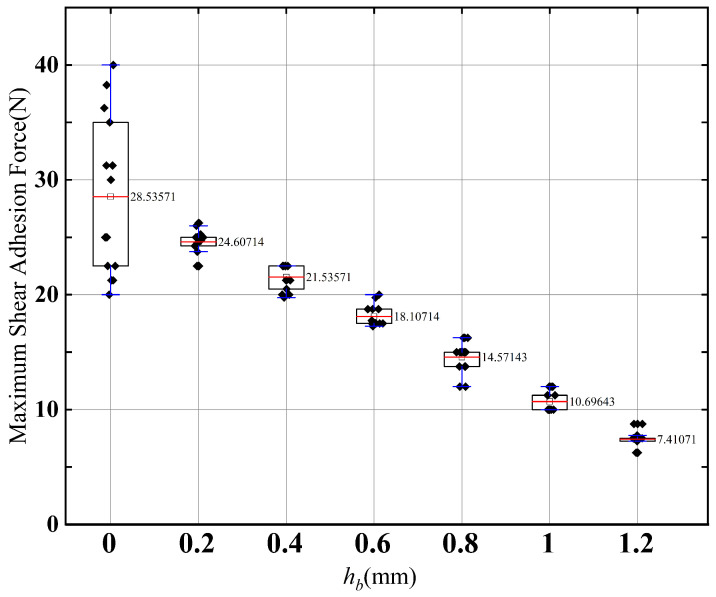
Relationship between initial cavity height and maximum shear adhesion force.

Since the initial volume of the cavity is changed in the pre-tensioning phase, the initial volume of the cavity determines the maximum shear adhesion force that the actuator can withstand. Therefore, in pre-tensioning phase, the volume of the cavity can be controlled by controlling the height of the cavity, thereby controlling the maximum shear adhesion force. The relationship between the cavity height change of the actuator and the maximum adhesion force is shown in Figure 10. In Figure 10, when the cavity height is 0, the dispersion of the maximum shear adhesion force is high. The reason for this is the uneven distribution of the preload resulting in incomplete contact between the cavity and the target surfaces.

We also planned several crawling gaits for the robot. One crawl cycle involves six steps in chronological order: (A) First, the body of the robot is lifted from the ground by actuating digital servos (DS1,3,5,7; Figure 8a(A)); (B) actuating DS2 to pull left forelimb forward (Figure 8a(B)); (C) actuating DS6 to pull right hindlimb forward (Figure 8a(C)); (D) actuating DS4 to pull right forelimb forward (Figure 8a(D)); (E) actuating DS4 to pull the left hindlimb forward (Figure 8a(E)); (F) resetting all of DS2, DS4, DS6, and DS8 to propel the body of robot forward (Figure 8a(F)). The process of locomotion begins to repeat from step (F). Then, repeating the above sequence steps (B to F) can achieve long distance movement (The speed of locomotion can reach 369 mm/min). When we adjust the actuating sequence of the digital servos (DSs), the robot can move laterally without turning.

To demonstrate potential of the robot to accomplish tasks by changing gait, we actuate the robot over an obstacle (Figure 8c): a stick 16 mm above the acrylic plate. In order to drive the robot over the obstacle, we use manual control to actuate the digital servos. In addition, manual control also simplifies motion planning. We actuate the robot to the obstacle with the simplified crawling gait, then raise its legs one by one over the obstacle, and finally return to its normal state. From the initial state (Figure 8a(A)), this sequence involves six steps. First, drive digital servos (DS1,3,5,7;) to elevate the body of the robot 3 cm from the ground on the basis of initial state (Figure 8b(B)). After elevating the body, raise its legs one by one over the obstacle with the gait mentioned above (Figure 8a(A–F)). Figure 8b shows the actuation sequence of the digital servos (DSs) that generates this locomotion.

Another significant advantage of the proposed climbing robot equipped with adhesion actuators is that the adhesion actuator relatively improves the load capacity of robot. When the robot crawls on the horizontal plane (Figure 8a), it can easily carry 500 g weight or even heavier objects. Furthermore, on an acrylic surface with a certain inclination, our robot can drag 500 g objects upwards (Figure 8b(A)).

### Experiments on Various Types of Target Surfaces

**Figure 11:**
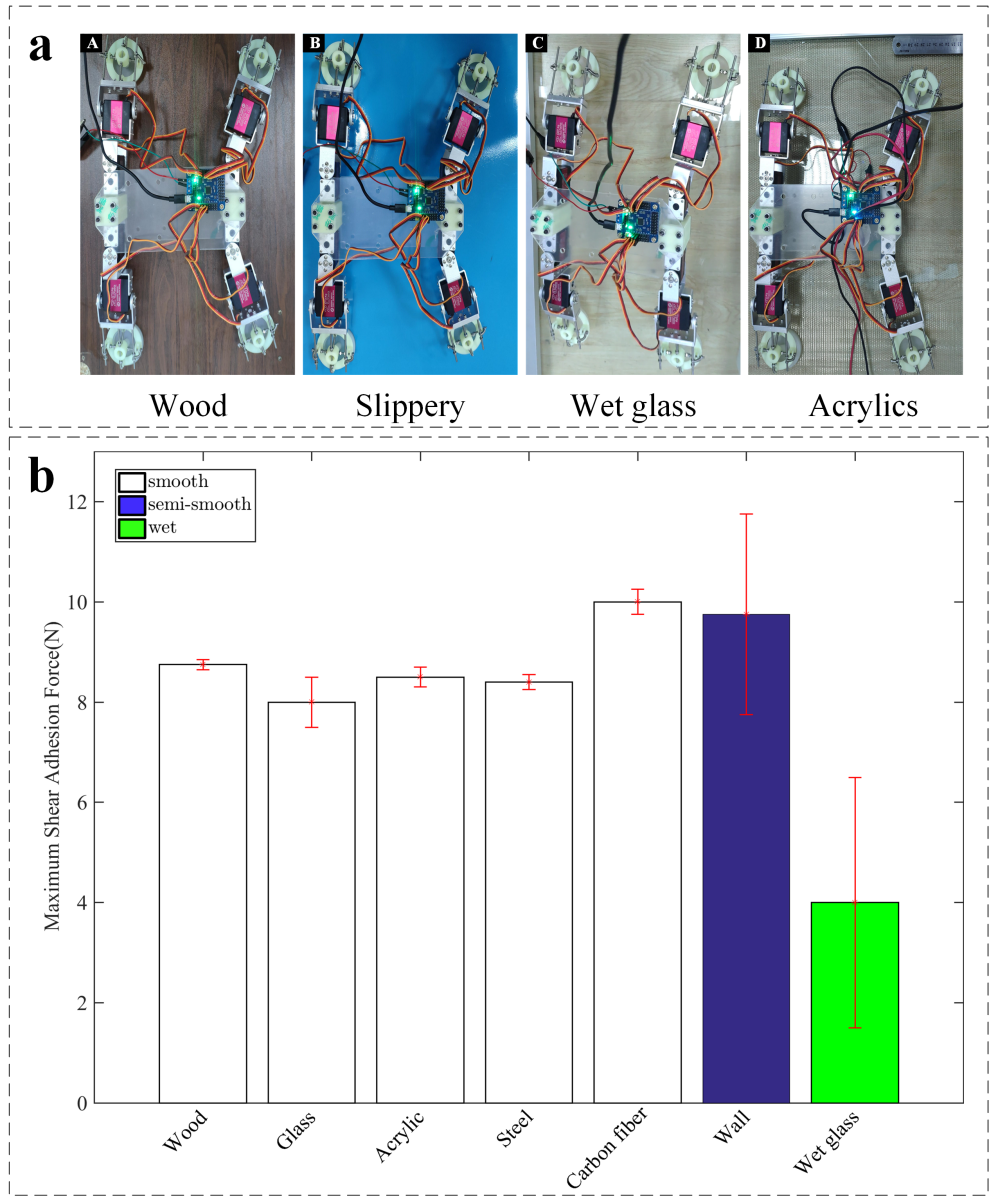
Characterization of adhesion force and demonstration of inclined crawling of robot on different substrates. (**a**) demonstration of the robot’s wide capability of climbing on different types of inclined surfaces with a carried 500 g load, (**A**) wood, (**B**) slippery steel, (**C**) wet class, (**D**) acrylics. (**b**) Results of the measured maximum shear adhesion force on different types of substrates.

To further evaluate its climbing performance, we test our robot on various types of target surfaces including semi-smooth surfaces, wet, or slip surfaces. Figure 11a illustrates that the proposed climbing robot has the ability to drag 500 g to crawl on various types of target surfaces, including dry, damp, smooth, and semi-smooth surfaces.

The maximum shear adhesion force $F_{S}$ of the adhesion actuator determines the maximum load capacity of the climbing robot. In order to further verify the load capacity and crawling capacity, we measure the maximum shear adhesion force $F_{S}$ generated by the actuator and various surfaces, including carbon fiber board, glass, wooden door, and steel. All measurements are made without additional load on the climbing robot. The results of measurements illustrate that the material of the target surfaces have little effect on the maximum shear adhesion force of the adhesion actuator. As illustrated in Figure 11b, the adhesion range of various measured target surfaces is 8 N to 10 N, demonstrating that the adhesion actuator has a strong load capacity and can carry objects several times its own weight to crawl on inclined surfaces with a certain angle.

As shown in Figure 11b, compared with the shear adhesion on the dry acrylics, the shear adhesion of the adhesion actuator to the wet acrylics is slightly smaller, but it is also large enough. Therefore, it can still work normally under harsh conditions such as glass walls and doors with water outside the building. The strong adhesion force demonstrated by the adhesion actuator of robot proposed in the experiments makes it more adaptable than passive suction cups of traditional robots when crawling on damp and slippery surfaces. Especially, because of the vertical load applied to the actuators, the proposed robot does not need to move all the time to maintain negative pressure like a robot equipped with passive suction cups.

Furthermore, we also test the possibility of the robot crawling on semi-smooth target surface. According to the classification of machined surface roughness, a semi-smooth surface can be defined as a surface whose arithmetic average height is greater than 12.5 μm and less than 25 μm. In order to prove the climbing performance on the semi-smooth surface, we take the painted wall ($R_{a}$ = 21 μm) as the experimental climbing test surface. The maximum adhesion force measured on the painted wall is about 9 N, which is relatively larger than the force measured on smooth surfaces. The friction of the semi-smooth surface is relatively larger than the friction of smooth surface, which is the reason for the higher shear adhesion force on the semi-smooth surface. In addition, as the contact area between the actuator and the target surface increases, the friction force increases and the shear adhesion force increases. Moreover, the error in the measured adhesion of actuator on painted wall shown in Figure 11b can be explained by the difference in the flatness of the painted wall.

## 4. Conclusions

In this study, we proposed a novel USAA for a climbing robot, which can carry a certain weight and crawl on a variety of inclined and horizontal target surfaces (smooth, semi-smooth, dry, or damp). We modeled the actuator adhesion as a double-layer doming system, and extracted essential design parameters. The relationship between the initial height of the cavity and the maximum shear adhesion force has also been studied, which is important for adjusting the initial height of the cavity according to the actual situation to obtain sufficient adhesion. The USAA broadens the design boundary of climbing robots, and has a wide range of potential applications in wall cleaning, bridge inspection, search and rescue, and reconnaissance. Although the USAA gets rid of the dependence on the vacuum pump, it still has certain limitations. An obvious disadvantage of the proposed climbing robot is that it can not transition from the ground to vertical surfaces because the focus of this study is the adhesion behavior of the proposed USAA, which is important for the gait and locomotion of the climbing robot. Furthermore, the speed of adhesion switching can not fully utilize the moving speed of the robot. Therefore, the future work is mainly to improve the USAA to increase the adhesion switching speed and optimize the motion mode of the climbing robot (such as switching between three-dimensional surfaces and crawling on surfaces with large curvature).

## Data Availability

The data presented in this study are available on request from the authors.

## Abbreviations

The following abbreviations are used in this manuscript:

USAA	Under-actuated soft adhesion actuator
